# Designing interventions to scaffold emotional awareness, beliefs and action tendencies

**DOI:** 10.1371/journal.pone.0335896

**Published:** 2025-11-03

**Authors:** Tanmay Sinha

**Affiliations:** National Institute of Education, Nanyang Technological University, Singapore; Mazandaran University of Medical Sciences, IRAN, ISLAMIC REPUBLIC OF

## Abstract

I demonstrate – via a novel intervention comprising evaluation of emotional contrasting cases and expressive writing – improvements in emotional awareness, beliefs and action tendencies among lower secondary students (*N* = 71). Relative to their pre-intervention profiles, students were able to better identify and articulate emotions, as well as showcase a more nuanced understanding of both positive and negative emotions and their respective roles in learning. While the intervention successfully reduced avoidance behaviors towards unpleasant emotions and encouraged a more balanced appraisal of emotional experiences, it also highlighted certain limitations – for instance, students showed little change in their acceptance of unpleasant emotions, suggesting further support may be needed to fully utilize emotions as learning tools.

## Introduction

Designing low-cost, scalable and ecologically-valid interventions that scaffold students’ sensemaking with emotions is an important yet underappreciated educational goal. Although impactful prior research efforts to foster emotional *awareness* exist (e.g., [1]), we do not empirically know yet if and whether students’ *beliefs* about the usefulness of emotions for learning and their *action tendencies* towards accepting or rejecting these emotions can be changed. Here, I take up the design challenge of offering one such novel intervention where students systematically evaluate *contrasting cases* as well as generate *expressive writing* excerpts, in the service of explicit emotional reflections within a safe classroom space.

### Theoretical background

#### Emotional awareness, beliefs and action tendencies.

*Emotional awareness* is defined as the ability to monitor, differentiate and evaluate one’s emotions and their physiological correlates [2] – its two key components include the ability to recognize and differentiate various emotions (e.g., sadness, anger, or fear), and bodily awareness, which refers to the physical sensations associated with emotional experiences. Emotional awareness has been empirically shown to contribute to adaptive emotion regulation across varied learning contexts [3]. Interventions to improve emotional awareness for adolescents, the targeted population, typically focus on mindfulness-based exercises (e.g., deep breathing, guided meditation), reflective journaling to help identify and articulate emotions, and empathy-building exercises to foster perspective taking, etc – see [4] for a review. I build on the idea of journaling in the present work via expressive writing, but incorporate novel reflection questions probing into metacognition about emotional usefulness for learning.

*Emotional beliefs* about usefulness pertain to an individual’s judgment regarding the helpfulness or hindrance of emotions in their lives [5]. For instance, even though a student may be able to recognize and articulate their feelings of sadness, they may simultaneously hold the belief that sadness is a harmful emotion to cultivate – this can, in turn, directly influence how sadness is approached (e.g., lead to avoidance) and utilized within a learning situation. Unfortunately, interventions for enhancing emotional awareness (e.g., [1]) do not always incorporate explicit strategies that encourage students to use both positive and negative emotions as tools [6] for personal growth and resilience. Given that emotions may serve not just hedonic but also instrumental goals [7], recent educational scholarship (e.g., [8]) has implicated designing new ways to foster improvements in students’ beliefs about the usefulness of emotions. That is precisely one key design goal in the present work, which I operationalize via contrasting cases.

*Emotional action tendencies* refer to the behavioral inclinations that arise in response to emotional experiences, influencing how individuals engage with their feelings by accepting or rejecting them [9]. For instance, tendencies to resist or suppress unpleasant emotions may lead to discrediting a valuable emotional learning experience and hinder adaptive coping mechanisms [7,8]. Conversely, fostering acceptance of pleasant feelings can enhance emotional well-being, but only if it doesn’t come at the cost of ignoring the downsides to maintaining an excessively positive façade [10]. Recognizing and cultivating balanced emotional action tendencies is crucial, yet there remains lack of pedagogical approaches deliberately designed to scaffold them.

Taken together, these three facets of emotional understanding – *awareness*, *beliefs* and *action tendencies* – are particularly critical during the developmental phase of adolescence when students face complex academic, social, and identity challenges [11]. Research has shown that students often have underdeveloped or inaccurate understandings of their emotions, which can negatively affect self-regulation, motivation, and learning outcomes [12,13]. For example, students may struggle to recognize or label emotions accurately, leading to maladaptive coping such as avoidance or emotional suppression, which inhibits effective engagement in school [14]. The present intervention is therefore deliberately *student-focused*, given the critical role of emotional skills in academic success and psychosocial development during the school years. While the foundational principles of emotional understanding are broadly applicable and could be adapted for general populations, I have designed this intervention with students to address the scaffolding needs for this demographic [15]. Here, I target students as a population rather than strictly by age, meaning the intervention components are tailored for educational contexts but may be applicable to young people spanning late childhood through adolescence.

#### Improving emotional awareness, beliefs and action tendencies.

Empirically, the evidence based on prior work is strongest for interventions focused on *emotional awareness* and regulation, with emerging but less direct research on *emotional beliefs* and *action tendencies*. Comprehensive social and emotional learning (SEL) programs are the most widely studied school interventions [16] – these brief *action-oriented* interventions can take several forms such as students learning to (i) recognize emotional patterns in themselves and others via active listening and analyses of emotional narratives, (ii) connect those patterns to bodily experiences via mindfulness and body awareness practices like deep breathing, mindful movement, (iii) reflect on emotional highs and lows via writing gratitude lists and drawing pictures to express feelings, and (iv) participate in structured discussions about the role of emotions in decision-making via role-plays and collaborative problem-solving activities. Research syntheses suggest such universal SEL programs typically enhance students’ *emotional awareness*, including the ability to recognize, differentiate, and manage emotions, as well as related social-emotional skills, prosocial behaviors, and well-being across diverse populations and age groups, while mitigating emotional distress (e.g., [16,17]). Mentalization-based interventions (MBIs), which explicitly train students and teachers to reflect on their own and others’ mental states, have also demonstrated significant improvements in emotional understanding, empathy, and emotion regulation, with some evidence of sustained effects [18].

Direct interventions on *emotional beliefs*, specifically those about the usefulness of emotions, are less common in educational research. However, social-psychological interventions that address students’ mindsets and beliefs about emotions and learning have shown promise in shifting attitudes and improving academic and social outcomes [19]. These interventions often work by reframing emotions as useful signals or resources, but more research is needed to isolate effects on *emotional beliefs* per se. Two other classes of interventions – Rational emotive behavior therapy (REBT) and Acceptance and commitment therapy (ACT) – have also been used in prior work to target *emotional beliefs*. For instance, REBT interventions, which teach students “how to examine and challenge their unhelpful thinking which creates unhealthy emotions and self-defeating strategies and behaviors” [20, p. 198], have been shown to reduce irrational beliefs about emotions in high school students. Such irrational beliefs often take the form of musts, absolute should, have-tos and so forth (e.g., “I must never feel frustrated”, “If I fail at something important to me, then I should be a failure”). ACT interventions, on the other hand, target *emotional beliefs* through experiential exercises on psychological flexibility, defined as “the ability to be in the present moment with full awareness and openness to our experience and to take action guided by our values” [21, p. 12]. By encouraging students to more accepting of their emotional experiences, cognitive defusion (interpreting thoughts as thoughts, as opposed to facts), promoting mindfulness (present moment awareness, without judgement), use of self-*as*-context (detaching from unhelpful narratives about the self), scaffolding identification of personal values that can serve as a guide for action, and inspiring students to take steps guided by values even in the presence of difficult internal experiences, ACT interventions have been shown to develop accepting rather than rejecting stances towards emotional experiences [22].

Finally, research directly targeting *emotional action tendencies* in educational contexts is limited because of the very recent conceptualization of the construct. Some studies have explored interventions to improve emotional-action control, such as physiologically-grounded brain stimulation or behavioral training to override automatic emotional responses, with promising results in controlled settings [23]. Dialectical behavior therapy (DBT) may offer a promising alternative to influence *action tendencies* by teaching students to pause, reflect, and choose adaptive responses to emotions – specifically, DBT interventions assist students with learning to notice an urge that arises with each emotion (e.g., the urge to avoid when anxious), name these urges non-judgmentally, evaluate whether the emotion fits the current situation and whether following the urge would be helpful or harmful, and most crucially, practice deliberate opposite action (e.g., approaching others and engaging instead of withdrawing). Through repetition, prolonged exposure to DBT has been shown to shift students’ tendencies from avoidance and rejection of emotions towards acceptance and agreement [24], albeit with mixed results (e.g., [25]). How may we instead design brief resource-efficient interventions that holistically target *emotional awareness*, *beliefs* and *action tendencies*?

#### Contrasting cases.

Contrasting cases exemplify a constructivist pedagogical approach where students are provided opportunities to notice the similarities and differences between two perspectives on a learning situation. Originating from research in perceptual learning [26], these instructional materials help attend to information that may otherwise be overlooked. In the present intervention, I use contrasting cases, typically used to improve discernment of critical task features in inductive STEM learning (e.g., [27]), in a novel way to illustrate how different subjective evaluations or appraisals of the same underlying emotion (e.g., anger) may lead to distinct outcomes. Simply put, contrasting cases allow students to engage in comparative analysis to construct their own understanding of the situational utility of emotions across relatable learning contexts [7].

This reflective emotional practice can be posited to help students recognize and differentiate emotional triggers along with their distinct regulatory implications, in turn improving *emotional awareness*. Comparison of cases is also likely to result in re-evaluation of preconceived notions about emotions, especially as students reflect on the resonance of those emotional cases to their own learning journeys, in turn shaping their *beliefs* about the usefulness of emotions. For instance, a case demonstrating how anger can be harnessed to motivate assertive communication may challenge a student’s belief that anger is solely harmful. Finally, a contrasting case activity is also expected to modify *emotional action tendencies* by illustrating the behavioral implications of different emotional responses. By evaluating cases where their peers adopt either a suppressive or an accepting stance towards emotions, students can observe the consequences of these tendencies on emotional well-being as well as formal/ informal learning outcomes. Taken together, the sensemaking provoked by engagement in contrasting cases may begin equipping students to critically evaluate their own and others’ emotions, a critical competency for maintaining emotional well-being in face of challenges [28].

#### Expressive writing.

Decades of prior psychological work have shown that written emotional disclosure can help individuals cope with stress, enhance emotional well-being and improve self-awareness [29]. By focusing attention on details of memories that have been inhibited, undervalued or avoided, this writing process deliberately nudges labeling and acknowledgment of emotionally significant experiences. For adolescents, addressing difficult learning experiences via such reflective practices can foster emotional resilience and a sense of agency, as they recognize their ability to overcome obstacles to progress and explore the rationale for accompanying emotions in a safe space [30]. Young learners, for instance, lower secondary students, may however require scaffolding to guide them through their emotions and break the writing process into less overwhelming and manageable steps [31].

I design one such structured expressive writing component in the present intervention, where students are encouraged to focus on the most strongly felt emotions during a prior learning situation and describe not just their thinking but also physical sensations associated with those emotions. Identifying and labeling their emotions as part of the writing process can improve *emotional awareness*, allowing students to understand the nuances of their feelings (e.g., whether they feel anger versus frustration and why do they feel that way). Although not part of the typical expressive writing protocol [32], the present intervention further incorporates explicit opportunities for metacognition about the potential usefulness of emotions students experienced – understanding the purpose of emotions can shift students’ *beliefs* from labeling them as merely good or bad [33] and help develop appreciation for the complex and often paradoxical roles emotions play in learning and social interactions [8,34,35]. At the same time, nudging reflection on the normalcy of their emotions can promote *emotional acceptance*, in turn helping students (i) recognize that experiencing a range of emotions (positive or negative) is a natural part of learning, and (ii) reduce the likelihood of suppression, which has been previously shown to be a maladaptive emotion regulation strategy [36].

### Research questions

RQ1: How does students’ *emotional awareness* change post-intervention?

RQ2: How do students’ *beliefs about the usefulness of emotions* change post-intervention?

RQ3: How do students’ *emotional action tendencies* change post-intervention?

## Method

### Participants

After obtaining informed written consent from students and their parents, *N* = 71 lower secondary school students (grade 7, age 12−13) from a specialized independent school in Singapore participated. The sample comprised 67.61% males (*n* = 48), 16.9% females (*n* = 12), 2.82% (*n* = 2) not listed or preferred not to say, with 12.68% (*n* = 9) missing data for gender. Ethnicity-wise, I had 64.79% Chinese (*n* = 46), 8.45% Indian (*n* = 6), 14.08% (*n* = 10) not listed or preferred not to say, with 12.68% (*n* = 9) missing data for ethnicity. The majority of students (73.23%, *n *= 52) had scored 75% or more in their most recent standardized annual national examination for English. Their self-reported confidence in reading skills (*M* = 3.77, *SD* = 0.91, *max* = 5) and writing skills (*M* = 2.98, *SD* = 1.11, *max* = 5) at school were deemed sufficient for study completion. Data collection was carried out in February 2025. The study was approved by the ethics commission of Nanyang Technological University (IRB-2023–1040).

### Study design and learning materials

**Fig 1:**
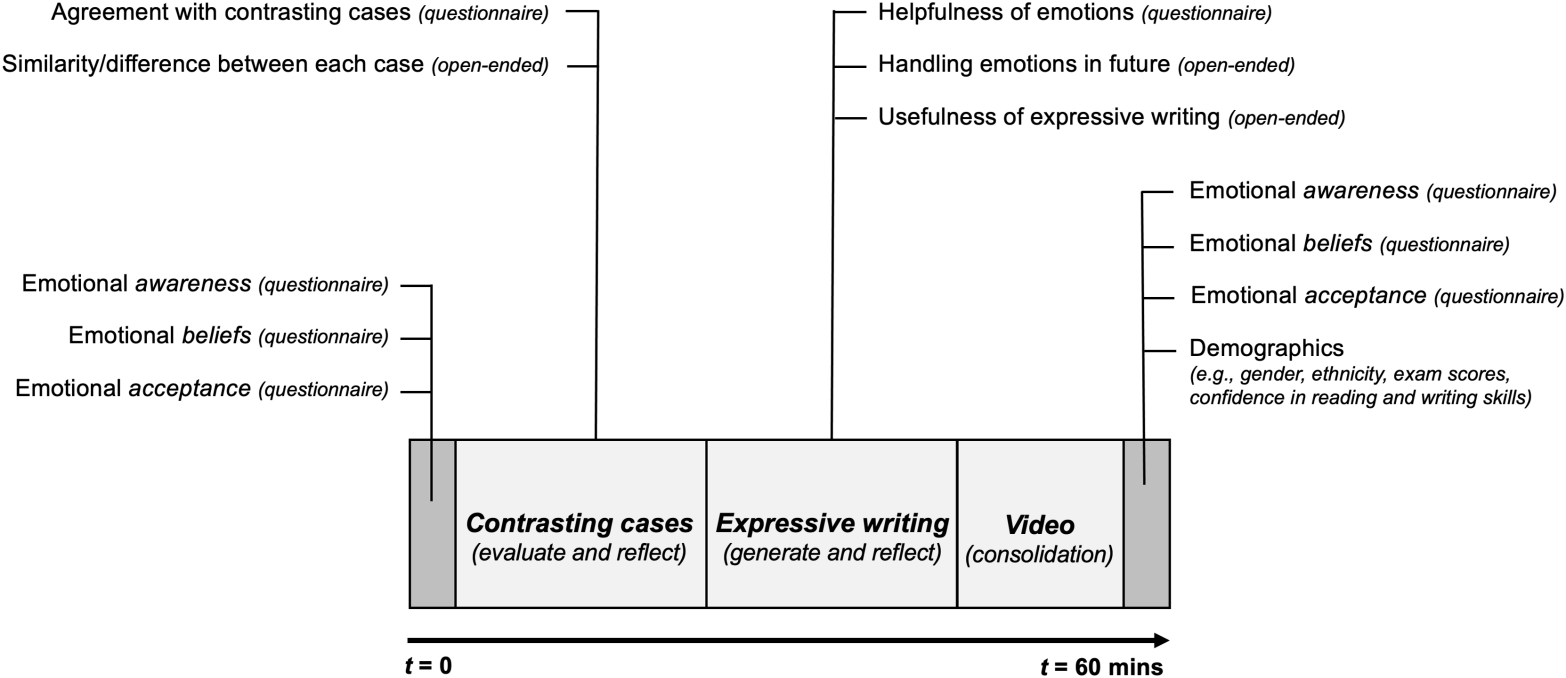
Study design.

I carried out a self-paced online study lasting up to one hour (*M* = 2032.22 seconds, *SD* = 554.54 seconds, *max* = 3592 seconds) during classroom time. See Fig. 1 for the study design.

Everyone began by evaluating six newly-designed pairs of *contrasting cases*, each focused on a formal or informal learning situation where students’ emotional reactions differed. Three negatively valenced cases focused on the self-conscious emotion of *shame*, the hostile emotion of *anger*, and the knowledge emotion of *confusion*, prototypical of challenging learning situations (e.g., [8,37,38]). The remaining three positively valenced cases covered the pleasurable emotion of *joy*, along with the corresponding self-conscious and knowledge emotion counterparts of *pride* and *interest* [39]. See Table 1 for two such contrasting cases (more examples under supplementary information S1), which show how the same emotion was appraised differently, resulting in seized or missed opportunities for learning. Students were tasked with assessing which of the two emotional reactions for each case resonated more with them – following each case, I therefore asked them to rate the extent to which they agreed with how the emotion was handled (five-point Likert scale ranging from *strongly disagree* to *strongly agree*). To push deeper reflection beyond superficial reading of the cases, the materials further probed students to identify and articulate one similarity and one difference between the two cases, focusing on the emotional reaction being showcased.

**Table 1:** Two exemplar contrasting cases focused on shame and pride in learning.

	Case #1	Case #2
Shame	*As I sat in the classroom, my heart raced when I realized I had forgotten to study for the math test. I was sure I would fail, and my cheeks turned red with* ***embarrassment*** *as I thought about letting everyone down. When the teacher handed back the tests, I saw my low score and felt a wave of* ***shame*** *wash over me. “Can I get some help?” I asked. My teacher smiled kindly, and I felt relief wash over me. I learned that asking for help is part of learning, and sharing my shame gave me a chance to grow. I promised myself to prepare better next time and felt proud for facing my feelings instead of running away from them.*	*When the teacher handed back our math tests, the* ***shame*** *hit me like a cold wave. I had studied hard, but my score was still low. I wanted to disappear, feeling like everyone was judging me. Instead of talking to my friends about how I felt, I sulked at lunch, letting my* ***embarrassment*** *twist into anger. “I’ll never be good at math,” I thought sadly. My* ***shame*** *made me pull away, convincing me that I was a failure. I missed the chance to connect with others who might have felt the same. Instead of learning from my mistakes, I let shame isolate me, deepening my frustration.*
Pride	*When my science fair project won first place, I felt* ***pride*** *swell inside me, especially after facing challenges during the project. I had struggled to gather information and build my model, overcoming many obstacles along the way. Instead of celebrating with my classmates, I started to think I was the best scientist ever. I ignored others’ ideas, thinking I didn’t need anyone’s input. My* ***pride*** *made me lazy, and I stopped trying to learn and grow. I let my success build a wall around me, thinking I was above working with others. In the end, I realized that my* ***pride*** *kept me from making valuable friendships and learning opportunities. As I enjoyed my success, I didn’t notice how my attitude pushed my peers away. Instead of building connections, I created walls that kept me from seeing the great talents around me.*	*After weeks of hard work on the school garden project, I felt so* ***proud*** *when we unveiled it. We faced challenges like bad weather and plants that wouldn’t grow, which made the project difficult at times. But we didn’t give up. When everyone gathered to see our garden, the bright colors of the flowers and the fresh vegetables made me happy. When I looked around at my classmates’ smiles, I realized that our teamwork had created something special. This* ***pride*** *motivated me to keep helping our school community. I learned that* ***pride*** *can be a powerful force for good when shared with others, and it inspired me to keep working together. Seeing our efforts come together made me grateful for my classmates. This project showed me that we can achieve beautiful things when we work as a team.*

Subsequently, everyone engaged in a structured *expressive writing* activity where they were nudged to recall a prior time when they had faced a failure or challenge at school or camp activity (up to 5 minutes). Students first spent time on sharing an overview of such an event, when it took place, and people who were part of this experience with them. Aligning with the protocol for validated expressive writing interventions [32], I provided explicit instructions for students to not worry about spelling, grammar, or punctuation and just write what comes to mind. The protocol also reminded everyone that the study was a safe space for them to express their feelings and that honesty was more important than perfect writing. After this initial warm-up, students engaged in an emotion brainstorming phase (up to 20 minutes) where they were asked to create a list of emotions felt, and for two emotions that they felt the strongest, share why they felt this way and what thoughts ran through minds as they experienced the two emotions. I further probed students to describe what they felt in their body (e.g., tension, warmth) and any physical reactions (e.g., tears, clenched fists). Finally, everyone was asked to try articulating how those emotions might be helpful and what this teaches them. The last phase of expressive writing (up to 5 minutes) asked students two foresight questions focused on (i) imagining facing a similar challenge or failure as their recalled event, and writing about how they would handle emotions better, (ii) articulating how might writing about emotions help them the next time a learning situation is hard (perceived usefulness of expressive writing).

After students engaged in both the *contrasting cases* and *expressive writing* intervention segments, a newly prepared consolidation video was presented (5 minutes). This consolidation stressed the importance of emotional resilience in learning by recapping key messages from each of the six contrasting cases, along with the relevance of expressive writing activities. As an attention check, five questions based on the video were designed as an immediate follow-up. See supplementary information S2 for details. Drawing on research syntheses [40,41] within the broader pedagogical scholarship of *sensemaking prior to instruction*, I conjectured that *evaluating* contrasting cases could scaffold students for the more cognitively demanding activity of *generating* expressive writing excerpts by building foundational understanding and awareness of key emotional features like valence, usefulness, etc. More generally, *evaluation* as a sensemaking activity primarily involves interpreting, comparing, and critiquing existing information to build a clearer understanding, which places a relatively lower cognitive load; by contrast, *generation* involves constructing new content or explanations, demanding higher-order thinking processes such as synthesis, creativity, and articulation of personal understanding [42,43]. Following scaffolding principles that support cognitive preparation and gradual complexity increase [44], the distinct cognitive demands of these two activities thus led to the sequential design decision of placing the *contrasting case*s activity before the *writing* activity.

### Pre-post measures

I collected pre-post measures of three key outcomes relevant to the study, all on a five-point Likert scale ranging from *strongly disagree* to *strongly agree*. First, *emotional awareness*, via the shortened form of a validated 10-item questionnaire adapted from [2] capturing items related to differentiating emotions (6 items, e.g., *“I am often confused or puzzled about what I am”*, *“It is difficult to know whether I feel sad or angry or something else”*) and bodily awareness (4 items, e.g., *“When I am scared or nervous, I feel something in my tummy”*, *“When I feel upset, I can also feel it in my body”*). Second, *emotional beliefs*, via a validated 8-item questionnaire adapted from [5] capturing the two distinct subdimensions of the usefulness of positive emotions (4 items, e.g., *“Positive emotions are very unhelpful to people”*, *“Positive emotions are harmful”*) and negative emotions (4 items, e.g., *“There is very little use for negative emotions”*, *“People don’t need their negative emotions”*). Third, *emotional acceptance*, via the shortened form of a validated 16-item questionnaire adapted from [9] capturing four distinct subdimensions of emotional action tendencies – rejection of unpleasant emotions (5 items, e.g., “*I try to resist unpleasant feelings as much as I can”*, *“I fight against my unpleasant feelings”*), rejection of pleasant emotions (4 items, e.g., *“I try not to feel pleasant feelings completely”*, *“I have gotten used to suppressing pleasant feelings”*), acceptance of unpleasant emotions (4 items, e.g., *“I usually allow myself to accept unpleasant feelings”*, *“I let unpleasant feelings happen”*), and acceptance of pleasant emotions (3 items, e.g., *“I can relate well to pleasant feelings”*, *“I allow myself to perceive pleasant feelings”*). Based on the data sample, all three scales showed good reliability pre-study (Cronbach’s α = 0.81, 0.72, 0.71) and post-study (Cronbach’s α = 0.84, 0.82, 0.84). See supplementary information S3 for complete scales.

The study concluded by capturing demographic data such as confidence in reading and writing skills (five-point Likert scale ranging from *strongly disagree* to *strongly agree*), scores from the most recently conducted primary school leaving examination, gender, and ethnicity.

### Analysis plan

Owing to the non-normal nature of the data (confirmed via a significant Shapiro-Wilk assumption check), a two-tailed paired samples Wilcoxon signed-rank test was used for evaluating pre-post changes in *emotional awareness*, *beliefs* and *action tendencies*. Rank-biserial correlation (r_rb_) was used as the non-parametric effect size measure to quantify the practical significance of the results. In cases of non-significant results, I used Bayes factor (BF_01_) to quantify strength of evidence favoring null hypothesis. All reported qualitative analyses followed a deductive approach to iteratively generate thematic codes [45], given that I already had a guiding theoretical framework underlying the study design and data analysis – for instance, (i) to evaluate implementation fidelity of expressive writing, these thematic codes focused on *emotional differentiation* and *bodily awareness*, and (ii) to evaluate perceptions of emotional helpfulness, these thematic codes focused on revealing shifts in students’ *appraisals of positive emotions* or *negative emotions*, both aligning with the expectations from measures administered during this intervention segment (see Fig 1).

## Results

### Fidelity and evidence of learning during the intervention

To evaluate whether students learned through interacting with the emotional *contrasting cases* as intended, I looked at whether they were able to discern the light and dark side of each emotion. Simply put, I evaluated their agreement with the case where an emotion was handled better (e.g., case #1 for shame and case #2 for pride, Table 1) and compared if it was higher than the contrasting case where the emotional scenario resulted in an undesirable outcome such as disengagement from the learning situation (e.g., case #2 for shame and case #1 for pride, Table 1). Across each of the six emotions (all *p*’s < 0.001, with r_rb_’s spanning 0.87–1), I found that students significantly agreed more with the case showcasing situations where emotions were leveraged as learning opportunities (*M* = 4.07, *SE* = 0.11 for anger; *M* = 4.04, *SE* = 0.12 for shame; *M* = 4.30, *SE* = 0.09 for confusion; *M* = 4.44, *SE* = 0.08 for joy; *M* = 4.38, *SE* = 0.09 for pride; *M* = 4.35, *SE* = 0.08 for interest) than when the emotional experiences from the cases resulted in missed opportunities for learning (*M* = 1.93, *SE* = 0.13 for anger; *M* = 2.17, *SE* = 0.13 for shame; *M* = 1.97, *SE* = 0.12 for confusion; *M* = 1.77, *SE* = 0.12 for joy; *M* = 1.73, *SE* = 0.12 for pride; *M* = 1.84, *SE* = 0.11 for interest).

**Fig 2:**
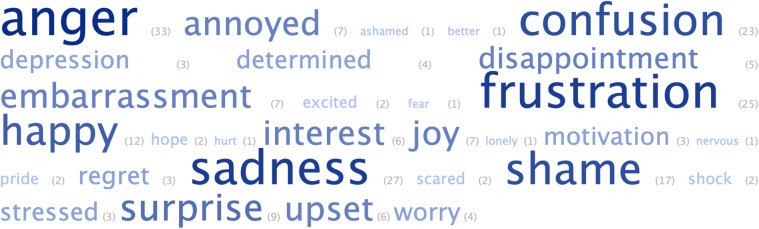
Word cloud of emotions and their frequencies reported during expressive writing.

To evaluate whether the *expressive writing* component of the intervention worked as intended, I first looked at total word counts during the writing process, and found that on average, students described their emotional experiences in fair detail (*M *= 108.50 words, *SD* = 64.27 words, *max *= 352 words). Fig 2 showcases a word cloud distribution with students’ most frequently recalled emotions as part of their writing excerpts. Exemplary thematic quotes from students highlighted their (developing) abilities to effectively differentiate emotions while participating in the writing task – for example, one student expressed self-doubt by stating *“I was confused as I studied extremely hard this time, yet achieved the lowest score I have ever gotten”*, and another reflected on their feelings of exclusion *“I felt this way as I was excluded from group work as no one wanted to be with me in a group…”*. Other students conveyed frustration with their peers, saying, *“I was angry as my group mates did not follow instructions and were not on task”*, while some shared their determination to improve *“…I was also determined because I wanted to try history since it was my first time and I told myself I needed to succeed…”*. Students further articulated bodily awareness of their emotions, for instance, by noting *“I had a few tears, I felt tense and rigid and my body felt really hot”*, or, *“I felt sad and my fists were clenched because I needed to find a way to improve”*. Taken together, the depth and specificity of these emotional reflections demonstrated meaningful engagement with the expressive writing intervention segment, indicating reasonable level of implementation fidelity. Qualitative evidence further confirmed that students were able to articulate how expressive writing can help them with emotional release and gaining more clarity into their emotional states (e.g., *“Writing helps me kinda spill all my emotions out, so I won’t feel as bottled up”, “It might help me remember about what had happened and help me to try not to repeat it again”*).

### Emotional awareness (RQ1)

Students significantly improved their emotional awareness after the intervention, with lower scores indicating better emotional awareness – pre-intervention (*M* = 2.56, *SE* = 0.09) and post-intervention (*M* = 2.45, *SE* = 0.08, *z* = 2.09, *p* = 0.036, r_rb_ = 0.30).

### Emotional beliefs (RQ2)

After undergoing the intervention, students appraised negative emotions as more useful, with lower scores indicating greater usefulness – pre-intervention (*M* = 2.41, *SE* = 0.09) and post-intervention (*M* = 2.13, *SE* = 0.09, *z* = 2.81, *p* = 0.005, r_rb_ = 0.42). At the same time, they appraised positive emotions as relatively less useful after the intervention, again with lower scores indicating greater usefulness – pre-intervention (*M* = 1.61, *SE* = 0.08) and post-intervention (*M* = 1.88, *SE* = 0.09, *z* = −3.20, *p* = 0.001, r_rb_ = −0.52).

### Emotional action tendencies (RQ3)

In terms of action tendencies towards unpleasant emotions, students showed a lower tendency to reject unpleasant emotions, reporting significantly lower post-intervention scores (*M* = 2.87, *SE* = 0.11, *z* = 4.70, *p* < 0.001, r_rb_ = 0.76) compared to pre-intervention scores (*M* = 3.41, *SE* = 0.10). However, they were not more likely to simply accept unpleasant emotions after undergoing the intervention (*M* = 2.99, *SE* = 0.10, *z* = 0.56, *p* = 0.57, r_rb_ = 0.09) relative to their pre-intervention reporting (*M* = 3.06, *SE* = 0.09), as the data sample showed strong odds supporting the null hypothesis (BF_01 _= 4.86).

When looking at action tendencies towards pleasant emotions, students showed lower tendency to simply accept pleasant emotions, reporting significantly lower post-intervention scores (*M* = 3.58, *SE* = 0.12, *z* = 3.26, *p* = 0.001, r_rb_ = 0.58) compared to pre-intervention scores (*M* = 3.90, *SE* = 0.11). At the same time, they showed similar tendencies to reject pleasant emotions before (*M* = 2.24, *SE* = 0.10) and after the intervention (*M* = 2.38, *SE* = 0.11, *z* = −1.87, *p* = 0.06, r_rb_ = −0.31), with weak odds favoring the null hypothesis (BF_01 _= 1.17).

## Discussion

I designed a novel intervention to scaffold emotional awareness, beliefs and action tendencies in lower secondary students. Everyone engaged in two primary learning activities – (i) evaluating and comparing emotional *contrasting cases* that showcased how positively and negatively valenced emotions might be less or more useful under different learning situations, and (ii) *expressive writing*, a generative activity where students engaged in structured reflection on a prior emotionally-charged learning experience and strategized how they might handle emotions better for future tasks. Manipulation checks showed that the intervention appeared to meet its goals. The sample provided evidence for moderate to strong effects for emotional outcome variables that changed significantly post-intervention (with r_rb_’s ranging from 0.30 to 0.76) – in terms of common language effect sizes, this represents up to a 77.48% chance that a randomly picked student post-intervention would have an improved emotional outcome relative to a student picked at random pre-intervention. In fact, posthoc analyses for the subgroup of students with poor(er) emotional awareness, beliefs and action tendencies (adjudged with a median split of self-reported scores in the pre-intervention data) consistently showed that the magnitude of improvements were greater relative to the entire sample for each of the outcome variables (see complete descriptive statistics under supplementary information S4). This indicates that the intervention had a stronger impact on those who may have needed it most. Although the limited number of items (typically, 3–10) used to measure the core constructs leaves open the possibility of average scores being sensitive to single-item changes, the observed changes, despite being small per individual, represent a consistent *group-level* effect. Drawing on [46], I argue that if the emotional sensemaking process triggered through the learning design were to affect this single individual (student) repeatedly over time, or, analogously, many individuals (e.g., students across multiple schools) simultaneously on a single occasion, one may expect the influences observed within this small sample to rapidly accumulate in their importance over time. Theoretically, the present work expands conceptualizations of what holistic emotional development during early adolescence may entail, and critically, how to intentionally design for guiding adolescents towards understanding the complexity of their emotional experiences, not only in terms of improved cognitive and bodily awareness but also more nuanced beliefs about usefulness of emotions and action tendencies for approaching them. These complementary sets of competencies are essential for developing the much-desired skill of *emotional resilience* [28], which may help to mitigate their disengagement and lack of persistence during challenging learning situations, regardless of cognitive abilities.

The results for improvements in self-reported *emotional awareness* (RQ1) indicate that students seemed to become more adept at identifying and articulating their emotions. This enhancement is an important precursor to regulating emotions [3]. When students develop the ability to differentiate between various emotions (e.g., confusion versus anger), this can potentially enable them to respond appropriately by engaging in reflective practices rather than reacting impulsively or resorting to avoidance behaviors/ maladaptive coping strategies [1].

The changes in students’ *emotional beliefs* (RQ2), particularly the shift in the appraisal of negative emotions as more useful, suggest a deeper understanding of the instrumental role that emotions play in learning [7]. This aligns with one of the intervention’s goal of illuminating the value of negative emotions, challenging the dichotomy that positions them as detrimental and positive emotions as inherently beneficial [47]. By recognizing the adaptive functions of emotions like shame and anger – such as motivating change or signaling unmet learning needs [8,35] – through vicarious learning from the emotional contrasting cases, students may have gained the ability to approach their emotional experiences with curiosity rather than valence-based judgments. At the same time, the results also showed a decrease in the perceived usefulness of positive emotions, indicating that students might have become more aware of their potential drawbacks – while positive emotions are generally considered beneficial in an idealized view [48], a more realistic stance recognizes that they can also lead to complacency or overconfidence, resulting in avoidance of necessary effort and underestimation of learning challenges [10,34]. The contrasting cases deliberately illustrated learning scenarios where the consistent pursuit of positive emotions could result in disengagement from reality (making it harder to cope with challenging but necessary tasks), and that the pressure to constantly display positivity might take an emotional toll when one is genuinely struggling at learning tasks [37]. An inflated sense of positivity can further make students less likely to acknowledge problems, identify areas for improvement and seek constructive feedback to engage in self-correction [49].

Taken together, these changes in self-reported appraisals of the usefulness of negative and positive emotions suggest that a short intervention such as this may have begun reconfiguring students’ conventional understandings (conflating emotional valence with usefulness) towards more balanced *emotional beliefs*, suggesting an overall maturation in their emotional understanding. Table 2 showcases exemplar thematic quotes from student reflections when asked how might positive or negative emotions be helpful and what this teaches them. Note that these student reflections were administered after the *contrasting cases* and *expressive writing* intervention segments and preceded the consolidation video presentation – they may therefore depict the emotional sensemaking process students engaged in prior to explicit telling. This data triangulation further complements students’ self-reported numerical changes in outcomes and shows evidence of meaningful shifts in perspectives about their emotions.

**Table 2:** Reflections signaling the learning process underlying improved emotional beliefs.

Shifts in the appraisal of negative emotions
*It’s okay to feel negative emotions as you need to let it out sometimes, it can help others understand you better – what makes you upset. Then they can learn from that and make sure to not do that to you.* *It is okay to feel angry and confused sometimes as they can help us shape our character and learn to control ourselves.* *I think it’s okay to feel angry at some things but not to the point where you start affecting other people around you.* *It’s ok to feel angry as it’s part of our learning. If we are never angry, we can’t learn well.* *It is ok to feel embarrassed as they are part of our lives and we need them. Sometimes they will make us feel arrogant and break connections. This teaches us to have emotions at the correct time.* *I felt it is ok to feel angry and shameful sometimes. Some of the emotions you feel initially could feel negative, but overtime, you learn from those mistakes and how you can cope with your feelings better.* *It is okay to feel regret as it allows you to think about what you did wrong and avoid making similar mistakes in the future.* *It is okay to feel worried as it shows that you care for somethings and will make sure that thing or someone will not get hurt or taken.*
**Shifts in the appraisal of positive emotions**
*It’s not okay to feel positive emotions sometimes as they can make you feel too high and start self-sabotaging your relationships with others.* *It’s sometimes not okay to feel happy and show it when the people you’re around are sad, might make them feel bad.* *I feel it’s not okay to be happy when it makes you ignorant.* *It is also not okay to feel proud or happy at other times as you may think that you are too good and may not put in as much effort as before.* *We cannot be too proud as it may seem like we are boasting. Taught me to work hard.*

This intervention appeared to impact *emotional action tendencies* (RQ3) in a notable way. The reduction in students’ self-reported tendency to reject unpleasant emotions indicates a movement away from avoidance behaviors, which are often counterproductive and can exacerbate emotional distress [50]. The intervention components might have contributed to normalizing the prevalence of unpleasant emotions in learning and showcased that they can be managed and do not have to be fought as hard. By not simply dismissing uncomfortable emotions, students in the study seemed to demonstrate an increased willingness to confront and process them. However, the lack of change in the acceptance of unpleasant emotions points to an area for further development – while reducing avoidance is a step forward, it is equally important for students to cultivate a mindset that welcomes unpleasant feelings as valuable experiences that can lead to insight and long-term growth [8,33], rather than always prioritizing feeling good in the short-term [7]. In other words, although students are less likely to resort to avoiding or suppressing unpleasant emotions, they may not have learned to fully embrace them yet, given that using unpleasant emotions as tools is presumably counter-intuitive and time-taking – for instance, prior research [6] implicates that learning to view unpleasant emotions as meaningful or growth-promoting may require repeated exposure to acceptance-oriented skills and additional scaffolding, such as guided reflection or experiential exercises. Proactively fostering this deeper shift is likely a longer-term developmental task that extends beyond the time frame of this work.

On the other hand, the results also showed a decline in the mere acceptance of pleasant emotions post-intervention, signaling that students might have become less inclined to accept such emotions whole cloth. Although the reduced acceptance of pleasant emotions may seem counterproductive at first because such emotions are often assumed to be unequivocally beneficial, in educational contexts this shift could represent a more discerning and adaptive orientation – when students approach pleasant emotions with greater critical awareness, they are less likely to automatically accept or cling to fleeting positive states, that is, prioritize feeling good at the expense of engaging with academic challenges [10,37]. This recalibration may help students avoid potential pitfalls associated with overvaluing positivity, such as complacency, unrealistic optimism, or a pressure to maintain constant positivity [34]. Fostering such a nuanced stance towards pleasant emotions may, in turn, promote emotional flexibility and persistence, enabling students to remain engaged even when authentic learning tasks evoke discomfort or frustration. In essence, rather than diminishing emotional well-being, then, a moderated acceptance of pleasant emotions may be indicative of emerging emotional maturity that can support longer-term growth within educational settings [7].

### Implications

The present resource-efficient psychological intervention holds several concrete implications for educators to cultivate an emotionally literate student population – first, establishing norms that position classrooms as a safe space for emotional expression and acceptance is key; second, inviting deliberate reflection and critique of students’ naturally occurring emotional experiences can deepen their understanding of emotion regulation; third, and most critically, designing lessons that explore the usefulness of positive and negative emotions via contrasting case studies or embodied role-playing activities may afford opportunities for students to (i) understand the specific utility or information emotions convey rather than simply labeling them as good or bad, and (ii) practice flexible, situation-appropriate ways to respond to various shades of these emotions. To be clear, I am not advocating for students to suppress or force emotions, but to navigate them wisely based on relevance to learning goals, irrespective of their valence.

### Limitations and future work

A few limitations of the present validatory work are worth noting. First, the three emotional outcomes were measured immediately after the intervention and may further require using correlational evidence with longer-term (delayed) outcomes and student behaviors to establish how long the reported effects can last. Second, the nuanced cultural context of the present intervention – where the collective is highly valued (e.g., group harmony and social norms), but there are strong individualistic tendencies (e.g., meritocracy, competition) too – may have lent itself relatively easily to achieving a balanced view of positive and negative emotions (upon high fidelity implementation). However, I conjecture that replicating this intervention within cultural contexts that favor predominantly (positive) emotional expression may generate data showing a greater resistance towards achieving balanced emotional states. Testing this hypothesis to strengthen the generalizability of this work will be an important future direction.

Third, given the time constraints that come with carrying interventions within the ecologies of authentic classrooms, the present work could not directly target more complex dimensions of emotional awareness (e.g., analyses of emotions, verbal sharing of emotions, attending to others’ emotions) beyond the foundational capacities of recognizing (through bodily cues), naming and distinguishing emotions (e.g., *shame* versus *anger* versus *confusion*). This led me to incorporate a narrower set of measurements that most closely aligned with the focus of the intervention. Future work could build on the intervention to prioritize the design of (and assessment for) higher-order cognitive processing of emotions (e.g., understanding *why* one feels a certain way, using feelings to understand events). Fourth, and finally, the present intervention does not allow making a causal claim about which of the two intervention components – contrasting cases, or, expressive writing – result in improved emotional outcomes. This exploratory work prioritized (i) ecological validity over causal inferences, (ii) capturing synergistic effects over isolating the impact of individual intervention components, and was primarily geared towards (iii) designing a holistic intervention package centered on sensemaking with emotions, which can offer more than one teaching route for practical implementation in everyday classrooms. Follow-up experimental work can test specific affordances of each intervention component in a more controlled manner and work towards refining theoretical models of emotionally-aware learning.

At the same time, establishing correlations of *emotional outcomes* I studied with *content-focused* outcomes would seem like a natural next step, aligning with a typical instrumentalist approach to the role of emotions in learning. However, contemporary learning sciences scholarship has begun conceptualizing emotion not just as an antecedent or lever for engagement or motivation for learning, but *as* learning [51]. I concur with this emerging stance in the field, emphasizing the need to appreciate emotional outcomes as a vital focus of research in and of themselves.

## Supporting information

S1 File Manuscript_SI_scales and learning materials. (PDF)

S2 File Manuscript_SI_data_anonymized. (XLSX)
