# Supplementary material for: Designing interventions to scaffold emotional awareness, beliefs and action tendencies
Source: PLoS One. 2025 Nov 3;20(11):e0335896. doi: 10.1371/journal.pone.0335896 (PMC12582459; doi:10.1371/journal.pone.0335896)
Supplement: S1 File — (PDF) [file pone.0335896.s001.pdf]

SUPPLEMENTARY MATERIALS

S1. *Contrasting case* intervention materials

	Case #1	Case #2
Shame	*As I sat in the classroom, my heart raced when I realized I had forgotten to study for the math test. I was sure I would fail, and my cheeks turned red with **embarrassment** as I thought about letting everyone down. When the teacher handed back the tests, I saw my low score and felt a wave of **shame** wash over me. "Can I get some help?" I asked. My teacher smiled kindly, and I felt relief wash over me. I learned that asking for help is part of learning, and sharing my shame gave me a chance to grow. I promised myself to prepare better next time and felt proud for facing my feelings instead of running away from them.*	*When the teacher handed back our math tests, the **shame** hit me like a cold wave. I had studied hard, but my score was still low. I wanted to disappear, feeling like everyone was judging me. Instead of talking to my friends about how I felt, I sulked at lunch, letting my **embarrassment** twist into anger. "I'll never be good at math," I thought sadly. My **shame** made me pull away, convincing me that I was a failure. I missed the chance to connect with others who might have felt the same. Instead of learning from my mistakes, I let shame isolate me, deepening my frustration.*
Anger	*When I got my science project back, I was really **angry** because I had put in so much effort, yet my teacher said it didn't meet the requirements. I felt like I had failed despite my hard work. I had thought I understood everything, and I was devastated to hear the criticism. I slammed my pencil down in frustration, letting my anger boil over. Instead of asking for feedback, I blamed my group for not helping enough.*<br>*My **anger** blinded me to the valuable lessons hidden in the feedback. I stormed out of the classroom, thinking the teacher didn't appreciate my effort. I missed the chance to learn and grow because I let my anger twist my thoughts, keeping me away from those who could have helped. In that moment of rage, I didn't see how my actions pushed my friends away. I could have realized we could support each other, but my **anger** created a barrier, making me feel even more alone.*	*After our team lost the basketball game, I was really **angry** because I felt my teammates weren't trying their best. We had practiced hard, and I thought we could win. After the game, I felt frustrated seeing my teammates shrug off the loss without discussing it. But instead of letting my **anger** take over, I decided to turn it into something good. "Let's talk about what went wrong," I suggested. My teammates agreed, and soon we were brainstorming ways to improve our skills for the next game. My **anger** turned into motivation, and I felt proud to be part of the team. It was a lesson in teamwork that I would remember, showing me how to change frustration into something positive. As we talked, I felt determined. I knew that if we worked harder, we could win our next game. This experience taught me that **anger** can help us improve when we use it the right way.*

Confusion	*When the science teacher introduced a new topic on ecosystems, I felt completely* ***confused****. The words were overwhelming, and the diagrams made no sense to me. I struggled to keep up and felt completely lost. Instead of speaking up, I stared blankly at my notes, thinking, "What's the point? I'll never understand this." I decided to give up and stopped participating in class discussions.*<br>*My* ***confusion*** *became a wall, blocking me from learning. I felt embarrassed and isolated, missing the chance to ask for help. By ignoring my* ***confusion****, I let it paralyze me instead of using it to learn more. Sitting quietly, I couldn't shake the feeling of being lost. The more I withdrew, the more I felt like an outsider, and the thought of asking for help seemed scarier than just staying* ***confused****.*	*In history class, we were discussing ancient civilizations, and I felt* ***confused*** *as the names and dates swirled in my mind. The lesson was moving quickly, and I struggled to keep up, especially when the teacher mentioned details I didn't understand. Instead of feeling lost, I raised my hand and asked questions. "Can you explain that again?" I asked, and my teacher's excitement helped clarify everything.*<br>*My* ***confusion*** *sparked curiosity, and I realized that asking questions is an important part of learning. Embracing that confusion opened a whole new world of understanding for me, reminding me that it's okay not to know everything right away. As I listened to my classmates' questions, I felt a sense of teamwork grow. We were all learning together, which made the experience exciting instead of scary.*
Joy	*When I won the art contest, I felt an overwhelming rush of* ***joy****, especially after struggling to get my artwork just right. I had faced challenges and self-doubt during the process of creating my piece. But instead of celebrating with my classmates, I started bragging about my talent. "I'm the best artist here!" I said, not realizing how it might hurt others. My* ***joy*** *turned into arrogance, making me blind to my friends' feelings. I isolated myself, thinking I didn't need anyone else. In my excitement, I forgot the importance of being humble and working together, which led to loneliness. As I enjoyed my victory, I couldn't see how my attitude pushed my friends away. Instead of creating connections, I built walls that kept me from appreciating the talents around me.*	*The day of the school talent show was amazing! I was nervous because I had practiced for weeks and wanted everything to go perfectly. When I stepped on stage to perform my dance, I felt a mix of fear and excitement. After a shaky start, I found my groove, and when the crowd erupted in applause, I felt pure* ***joy****. The cheers from my friends made me feel so happy, and I realized how much I love performing. That joy filled me with confidence and helped me appreciate the moment, showing me that sharing my passion is what really matters. I learned that* ***joy*** *can motivate us to chase our dreams and connect with others. After the performance, I felt like I was on top of the world, and I wanted to encourage my friends to share their talents too. It felt great to support each other in our successes.*

Pride	*When my science fair project won first place, I felt* ***pride*** *swell inside me, especially after facing challenges during the project. I had struggled to gather information and build my model, overcoming many obstacles along the way. Instead of celebrating with my classmates, I started to think I was the best scientist ever. I ignored others' ideas, thinking I didn't need anyone's input. My* ***pride*** *made me lazy, and I stopped trying to learn and grow. I let my success build a wall around me, thinking I was above working with others. In the end, I realized that my* ***pride*** *kept me from making valuable friendships and learning opportunities. As I enjoyed my success, I didn't notice how my attitude pushed my peers away. Instead of building connections, I created walls that kept me from seeing the great talents around me.*	*After weeks of hard work on the school garden project, I felt so* ***proud*** *when we unveiled it. We faced challenges like bad weather and plants that wouldn't grow, which made the project difficult at times. But we didn't give up. When everyone gathered to see our garden, the bright colors of the flowers and the fresh vegetables made me happy. When I looked around at my classmates' smiles, I realized that our teamwork had created something special.*<br>*This* ***pride*** *motivated me to keep helping our school community. I learned that* ***pride*** *can be a powerful force for good when shared with others, and it inspired me to keep working together. Seeing our efforts come together made me grateful for my classmates. This project showed me that we can achieve beautiful things when we work as a team.*
Interest	*When I joined the after-school robotics club, I was really* ***interested*** *in the projects. However, I faced the challenge of balancing my ideas with the group's. I became so focused on building my robot that I ignored group discussions. "I know what I'm doing," I thought, dismissing others' ideas.*<br>*My* ***interest*** *turned into an obsession, causing me to miss out on important teamwork lessons. I realized too late that working with my group could have made my project even better. My narrow focus led me to feel alone, keeping me from learning from my teammates and deepening my understanding of robotics. In my single-minded pursuit, I lost the chance to learn from my friends' unique ideas. I could have created something amazing together, but my self-absorption kept me from enjoying that collaboration.*	*On the first day of summer camp, my heart raced with excitement as I explored all the activities. I faced the challenge of stepping out of my comfort zone, especially when I saw some of the older kids already skilled at the arts and crafts. The art station caught my eye, and I eagerly joined in. I got to try painting and pottery for the first time! Each new skill I learned fueled my excitement and creativity. My* ***interest*** *in art grew, and I couldn't wait to share my creations with my family. This summer became a wonderful journey of discovery, helping me realize that following my interests leads to growth and connection. As I worked with others at the art station, I discovered new techniques and ideas, making the whole experience even better. I learned that sharing interests can spark even more creativity.*

SUPPLEMENTARY MATERIALS

S2. Consolidation *video*

Link to the video: https://www.youtube.com/watch?v=kQFrMPs8bRo

Follow-up attention check questions:

Question 1: What is emotional resilience, according to the video?
a) Ignoring your feelings
b) Only feeling positive emotions
c) Understanding, managing, and using your feelings to learn and grow
d) Avoiding difficult situations

Correct Answer: c)

Question 2: According to the video, can negative emotions be helpful?
a) No, negative emotions are always harmful
b) Yes, negative emotions can lead to important growth if we respond in a positive way
c) Only if you ignore them
d) Only if you share them with others

Correct Answer: b)

Question 3: In the video, what emotion did a student feel after getting a low score on a math test?
a) Joy
b) Anger
c) Confusion
d) Shame

Correct Answer: d)

Question 4: What is one way to manage emotions discussed in the video?
a) Ignoring your emotions
b) Expressive writing, like writing in a journal
c) Blaming others for your feelings
d) Avoiding new experiences

Correct Answer: b)

Question 5: According to the video, what can pride lead to if not managed carefully?
a) Increased motivation
b) Better teamwork
c) Arrogance and isolation
d) Greater happiness

Correct Answer: c)

SUPPLEMENTARY MATERIALS

S3. Complete *scales* used in the study

Emotional Awareness (5-point Likert scale from "strongly disagree" to "strongly agree")

1. I am often confused or puzzled about what I am feeling [*Differentiating Emotions* subscale]
2. It is difficult to know whether I feel sad or angry or something else [*Differentiating Emotions* subscale]
3. I never know exactly what kind of feeling I am having [*Differentiating Emotions* subscale]
4. When I am upset, I don't know if I am sad, scared or angry [*Differentiating Emotions* subscale]
5. Sometimes, I feel upset and I have no idea why [*Differentiating Emotions* subscale]
6. I don't know when something will upset me or not [*Differentiating Emotions* subscale]
7. When I am scared or nervous, I feel something in my tummy [*Bodily Awareness* subscale]
8. When I feel upset, I can also feel it in my body [*Bodily Awareness* subscale]
9. I don't feel anything in my body when I am scared or nervous [*Bodily Awareness* subscale] *(rev)*
10. When I am sad, my body feels weak [*Bodily Awareness* subscale]

Emotional Beliefs (5-point Likert scale from "strongly disagree" to "strongly agree")

1. There is very little use for negative emotions [*negative-usefulness* subscale]
2. People don't need their negative emotions [*negative-usefulness* subscale]
3. Negative emotions are harmful [*negative-usefulness* subscale]
4. The presence of negative emotions is a bad thing for people [*negative-usefulness* subscale]
5. Positive emotions are very unhelpful to people [*positive-usefulness* subscale]
6. There is very little use for positive emotions [*positive-usefulness* subscale]
7. People don't need their positive emotions [*positive-usefulness* subscale]
8. Positive emotions are harmful [*positive-usefulness* subscale]

Emotional Acceptance (5-point Likert scale from "strongly disagree" to "strongly agree")

1. I try to resist unpleasant feelings as much as I can [*reject unpleasant emotion* subscale]
2. I fight against my unpleasant feelings [*reject unpleasant emotion* subscale]
3. I try to suppress my unpleasant feelings as much as possible [*reject unpleasant emotion* subscale]
4. I try to push aside unpleasant feelings [*reject unpleasant emotion* subscale]
5. I try to bottle up unpleasant feelings [*reject unpleasant emotion* subscale]
6. I try to curb pleasant feelings [*reject pleasant emotion* subscale]
7. I try not to feel pleasant feelings completely [*reject pleasant emotion* subscale]
8. I have gotten used to suppressing pleasant feelings [*reject pleasant emotion* subscale]
9. I block out pleasant feelings [*reject pleasant emotion* subscale]
10. I permit myself to experience unpleasant feelings [*accept unpleasant emotion* subscale]
11. I usually allow myself to accept unpleasant feelings [*accept unpleasant emotion* subscale]
12. I let unpleasant feelings happen [*accept unpleasant emotion* subscale]
13. I allow myself to show unpleasant feelings [*accept unpleasant emotion* subscale]
14. I can relate well to pleasant feelings [*accept pleasant emotion* subscale]
15. I allow myself to perceive pleasant feelings [*accept pleasant emotion* subscale]

I accept pleasant feelings [*accept pleasant emotion* subscale]

SUPPLEMENTARY MATERIALS

S4. ***Quantitative analyses filtered by the subgroup with poor(er) emotional awareness, beliefs and action tendencies pre-intervention (based on median split of the entire pre-intervention data)***

Emotional Construct	Change, effect size, and sample size
Awareness	Pre-intervention – $M = 3.17$, $SE = 0.07$<br>Post-intervention – $M = 2.94$, $SE = 0.06$<br>($r_{rb} = 0.58$**) ($N = 35$)
Beliefs – negative emotions	Pre-intervention – $M = 3.02$, $SE = 0.11$<br>Post-intervention – $M = 2.34$, $SE = 0.13$<br>($r_{rb} = 0.86$***) ($N = 33$)
Beliefs – positive emotions	Pre-intervention – $M = 1.05$, $SE = 0.02$<br>Post-intervention – $M = 1.40$, $SE = 0.10$<br>($r_{rb} = -0.93$**) ($N = 34$)
Action tendencies – rejection of unpleasant emotions	Pre-intervention – $M = 4.21$, $SE = 0.08$<br>Post-intervention – $M = 3.22$, $SE = 0.18$<br>($r_{rb} = 0.95$***) ($N = 27$)
Action tendencies – acceptance of unpleasant emotions	Pre-intervention – $M = 2.46$, $SE = 0.09$<br>Post-intervention – $M = 2.60$, $SE = 0.11$<br>($r_{rb} = -0.34^{n.s.}$) ($N = 35$)
Action tendencies – acceptance of pleasant emotions	Pre-intervention – $M = 4.78$, $SE = 0.06$<br>Post-intervention – $M = 4.35$, $SE = 0.19$<br>($r_{rb} = 0.84$**) ($N = 26$)
Action tendencies – rejection of pleasant emotions	Pre-intervention – $M = 1.45$, $SE = 0.08$<br>Post-intervention – $M = 1.74$, $SE = 0.14$<br>($r_{rb} = -0.68$**) ($N = 31$)

Note: Wilcoxon's signed-rank test with paired samples has been used for pre-post comparisons. All self-reported scores on a five-pt. Likert scale (**: $p < 0.01$; ***: $p < 0.001$; n.s.: not significant).
